# Study on the optimization of temperature uniformity in the oven under the forced convection mode

**DOI:** 10.1038/s41598-023-39317-w

**Published:** 2023-08-01

**Authors:** Zongliang Yang, Dongxu Cheng, Bin Su, Chuang Ji, Jiayu Huang, Haiyun Li, Kai Zhang

**Affiliations:** 1 College of Metrology and Measurement Engineering, China Jiliang University, Hangzhou, 310018 China; 2 China Tobacco Henan Industrial Co., Ltd, Zhengzhou, 450000 China; 3 Gemac Engineering Machinery Co., Ltd., Xiangyang, China; 4 TK.CN INSURANCE CO. LTD., Beijing, China

**Keywords:** Electrical and electronic engineering, Fluid dynamics, Thermodynamics

## Abstract

In order to study the temperature distribution in a multi-functional oven and optimize the structural parameters of the oven, the internal temperature uniformity was improved. The test was conducted and the numerical simulation was conducted in two ways. The distribution of temperature field in each layer of ovens was measured in real time using a 13-point distributed thermocouple, and the oven temperature uniformity index was measured and analyzed. The temperature field inside the oven was numerically simulated using computational fluid dynamics. Investigated the heat conduction, convection and radiation effects respectively, and found out the main heat transfer mode of the oven. Further through the test of temperature measurement, verify the accuracy of the numerical simulation method. According to the results of the experiment and simulation, the reason of the uneven temperature field in the original structure of the oven was revealed and analyzed. By changing the structure of the oven tailgate, adjusting the air volume distribution, changing the distribution of the air outlet and other measures, greatly improving the uniformity of the temperature field inside the oven.

## Introduction

The development of new design trends in the kitchenware market is a dynamic process, which means that manufacturers need to develop and apply more advanced technologies. From coming up with an idea to bringing a new product to market, the R&D cycle becomes more urgent. Therefore, the time left for pre-research and product improvement is also relatively short. This shows that the way that takes more time to verify oven performance through experiment gradually cannot adapt to the new mode of product development nowadays. The application of numerical simulation can help developers to speed up the development process.

With the development of the national economy, oven cooking has emerged as an increasingly important trend in people's daily lives. The uniformity of the internal oven temperature is closely associated with the quality of baked goods. Non-uniform internal temperatures can result in uneven distribution of heat, leading to unsatisfactory coloration. Furthermore, uneven cooking renders the food inedible. Two methods are employed to investigate the internal temperature uniformity of ovens: experimental research and computer simulation. Experimental research offers the advantage of direct measurement and analysis within actual ovens, yielding reliable results. Meanwhile, computer simulation is a valuable tool for simultaneously simulating various conditions. Consequently, numerical simulation is widely applied in the study of airflow and heat transfer characteristics within ovens.

At present, there have been some researches on the thermal environment in oven-like equipment. For example, Yuan Hong et al.^1^ used computational fluid dynamics method to conduct numerical simulation of the internal temperature field of the oven, and revealed and analyzed the reasons for the non-uniform temperature field of the original structure of the oven. Lin How Chao et al.^2^ made an in-depth discussion on the distribution of internal temperature field of the oven and the method of oven temperature control system. Zheng Jinlong^3^ studied the heat distribution of baking trays in the oven, and used the Fourier heat conduction equation to explain that the heat distribution of baking trays of different shapes is different. Through measurement and CFD numerical simulation, Xiang Linlin et al.^4^ studied the distribution status of the temperature field in the inner cavity of an embedded oven, and proposed to improve the temperature uniformity by changing the hole in the top of the oven, the wind speed of the hot fan, the position of the heating tube, the structure of the hot fan and other optimization methods. Wang Jing et al.^5^ showed that when food temperature rose above 171 °C, convection became the dominant heat transfer method, and obtained an optimal oven model by using numerical simulation. Zhang Lanxin et al.^6^ simulated the internal temperature field of the oven model under different operating modes by means of numerical simulation. The research results showed that the uniformity of the internal temperature field of the existing oven model could be optimized by improving the oven structure. Gu Siyuan et al.^7^ simulated the influence of various improvement measures on internal cavity temperature distribution by establishing a three-dimensional simplified model of oven, and finally obtained the optimal model of oven by integrating various measures. Yao Jing et al.^8^ analyzed the problem of food heating in the oven from the perspective of mechanism, provided the thermal energy distribution of several typical food shapes during the heating process, and concluded that the circle was the optimal shape of the vessel through comparison. Li Baoqiang et al.^9^ proposed a control method based on fuzzy adaptive PID for the temperature control of pyrophyllite oven. By establishing a fuzzy control model, the three parameters of PID were corrected online. Tian Songtao et al.^10^ proposed four improved designs for the uneven distribution of velocity at each nozzle outlet in the original model. Through simulation analysis, it was concluded that the optimal simulation effect was achieved when the air distribution chamber was designed as a trapezoid and the nozzle arrangement was improved to be triangular. This paper takes the embedded electric oven of a certain manufacturer as the research object. According to the measured data of this kind of oven, the difference between the highest value and the lowest value of different measuring points in the oven cavity can reach 10 °C or more, which will affect the baking quality of food. In order to improve the performance of the electric oven in the actual use process, this study through the combination of experiment and numerical simulation, verify the temperature distribution of the oven cavity, so that the distribution is even.

## Experiment

In order to validate the established numerical model and assess the boundary conditions, experimental measurements were conducted. The testing standards for oven baking performance referenced EN 60350-1. The testing was divided into two steps. Firstly, the heating performance of the empty oven was measured, and temperature data were recorded for comparison with the simulated data. Subsequently, the oven underwent testing during baking, and temperature data as well as browning of the food were recorded. The testing encompassed temperature measurements and recordings during the baking process, along with post-baking surface browning. A cake baked on a baking tray was selected for testing as it serves as a good indicator of the oven's baking performance. Design flaws in the oven, such as uneven temperature or radiation distribution, are reflected in the browning of the cake. To study the actual temperature distribution inside the oven cavity, temperature measurements were taken on the intermediate surface of the oven. Thirteen measurement points were evenly distributed on the intermediate surface, as illustrated in Fig. 2. The testing instrument employed was an Agilent 34972A data acquisition system, recording data at a frequency of 1 s. The measurement range was 0–800 °C with an accuracy of 0.1 °C.

**Figure 1:**
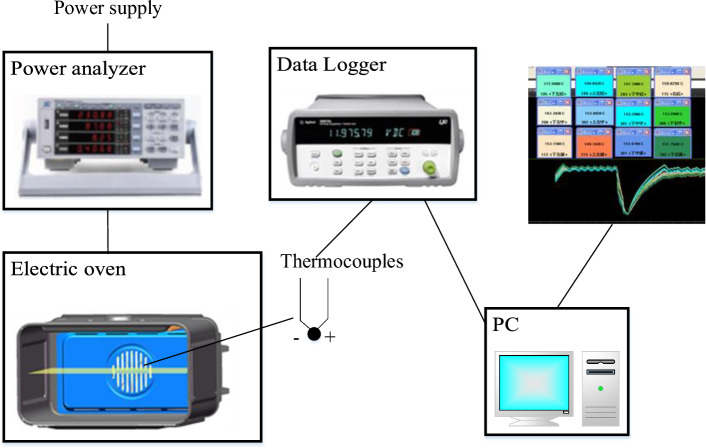
Equipment for the baking experiment.

**Figure 2:**
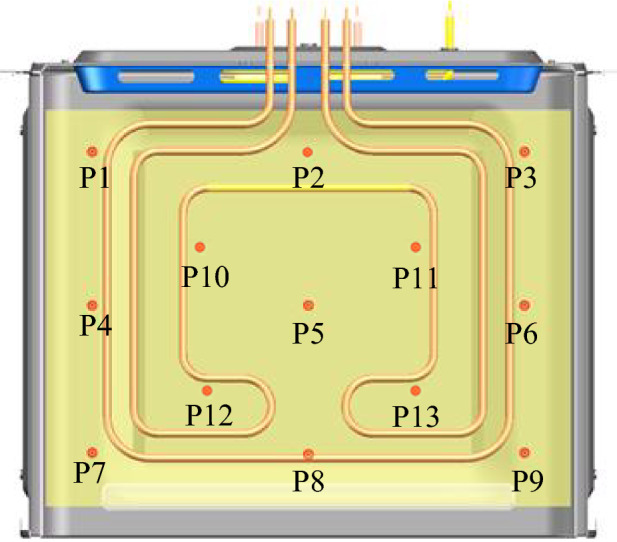
Locations of temperature measurement inside the oven cavity.

During the baking process, the temperature variation of the oven was recorded from room temperature to the set temperature over time. The temperature distribution was measured on a plane parallel to the bottom and located 140 mm above it. By evenly distributing 13 temperature sensors in space, the average temperature and standard deviation on this plane were obtained. The experiment utilized a 220 V AC power supply provided by a voltage regulator operating at a frequency of 50 Hz. Voltage, current, power, and consumption were monitored using an ISKRA MC740 digital power meter. A K-type thermocouple served as the temperature sensor on the measurement plane and was connected to the Agilent 34972A data acquisition device through a data acquisition card. The 34972A is a data acquisition device connected to a computer via a LAN interface, which converts the electrical signals from the thermocouple into temperature data and displays them on the computer screen. The experimental setup of all the devices can be seen in Figs. 1 and 2.

The baking performance of the oven was tested in forced convection mode and the oven temperature was set at 180 °C, indicating that the oven's circular heater and convection fan were operating simultaneously. The cake is made according to EN 60350:18.5% eggs, 29% flour, 15.8% corn oil, 18.5% milk and 18.5% sugar^11–13^. Mix the ingredients well and pour into a baking pan and place in the oven to bake. The length of the baking pan is 315 mm, the height is 45 mm and the width is 215 mm. The thermal parameters of the cake are shown in Table 1.

**Table 1:** Physical properties of the materials in use.

Materials	Thickness (mm)	*k* (W·(m·K)^−1^)	Cp (J·(kg·K)^−1^)	ε	ρ (kg·m^−3^)
Air ideal gas	–	0.0261	1004.4	–	Ideal gas
Glass	4	1.4	750	0.15	2500
Isolation	20	0.04	670	–	50
Cavity	0.8	45.7	513	0.9	6515.5
Heater	6.5	60.5	434	0.85	7854
Fan	0.8	60.5	434	0.85	7854
Fan cover	0.6	45.7	513	0.9	6515.5
Baking tray	0.6	45.7	513	0.9	6515.5

## Preprocessing of numerical calculation

### Structure of the oven

**Figure 3:**
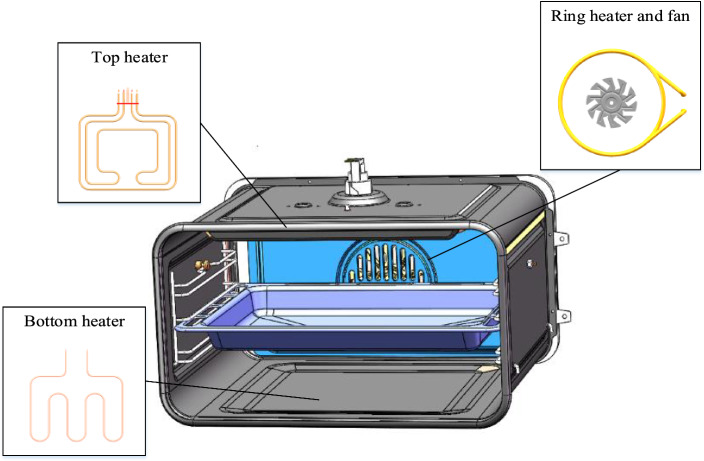
Schematic diagram of oven structure.

The pretreated physical model of the oven consists of two parts, the fluid domain and the solid domain. The fluid domain consists of the air inside the oven chamber. Solid domain contains oven insulation, door glass, fan cover, heater and baking tray^14–16^. The dimensions of the 3D model of the oven are 595 mm in width, 455 mm in height and 520 mm in depth. Model the entire fan assembly in SolidWorks with a fan diameter of 126 mm. Use a circular pattern to create 10 fan blades with an angular spacing of 36°. In air baking mode or fast heat mode, the fan rotates at 1350 RPM to enhance the effect of convection. The oven has three sets of heating devices: a circular heater at the back of the oven with a power of 1300 W, a heater at the top of the oven with a power of 2100 W, and a heater at the bottom of the oven with a power of 800 W. In simulation, their heat flux is specified to make them work. The unsteady state of 1500S was calculated by numerical simulation, and the time step was 5S. The schematic diagram of the oven structure is shown in Fig. 3.

### The governing equation

When simulating the baking process, the effects of convection, conduction, and radiation are taken into consideration. Definitions of boundary conditions refer to published research papers^17–19^ and experimental measurements. The air in the oven chamber is set as an ideal gas. According to the experimentally measured Reynolds number *Re* = 6.8 × 10^4^, it is assumed that the flow state of air is turbulence. Realizable k-epsilon turbulence model is used for numerical calculation because it improves simulation accuracy relative to the standard k-epsilon model. The heater generates high temperatures during the baking process, so the radiation effect plays a very important role in the simulation. Optical thickness is a good indicator for radiation model selection^20^. The P1 model and the Rosseland model are only suitable for optical thickness greater than 1, while the optical thickness of the oven L = 0.0045 is much less than 1. Only the Ordinates model can simulate the translucent material (the oven door glass). The following Reynolds averaged Navier–Stokes equations for flow and heat-transfer in the oven cavity were solved^21^.

The continuity equation:

$$\frac{\partial \rho }{{\partial t}} + \nabla \cdot \left( {\rho \vec{v}} \right) = 0$$

The Momentum governing equation:

$$\frac{\partial }{\partial t}\left( {\rho \vec{\nu }} \right) + \nabla \cdot \left( {\rho \vec{\nu }\vec{\nu }} \right) = - \nabla p + \nabla \cdot \left( {\overline{\overline{\tau }}} \right) + \rho \vec{g} + \vec{F}$$

The energy governing equation:

$$\rho c_{p} \frac{DT}{{Dt}} = k\nabla^{2} T$$

Radiation intensity conservation (DO):

$$\nabla \cdot \left( {I\left( {\vec{r},\;\vec{s}} \right)\vec{s}} \right) + \left( a \right)I\left( {\vec{r},\;\vec{s}} \right) = an^{2} {{\left( {\sigma T^{4} } \right)} \mathord{\left/ {\vphantom {{\left( {\sigma T^{4} } \right)} \pi }} \right. \kern-0pt} \pi }$$

where $\vec{v}$ is the air velocity field,$p$ is the pressure and *T* is the temperature. The density $\rho$ of the air was obtained by the ideal gas state equation, since due to high temperature differences in the oven, changes in air density are important. The thermal conductivity $k$ and the viscosity $\nu$ of air as well as the specific heat ${\text{c}}_{p}$ were considered to be constant. The solid material properties were also considered to be constant, as their variation with the temperature had a small effect on the baking process.

### The boundary conditions and simulation set-up

The analysis of the temperature field in the oven is based on three-dimensional, incompressible, steady-state flow and heat transfer simulations. The standard wall function is used to solve the boundary layer grid, and the DO (Discrete Ordinates) radiation model is selected based on the suitability of the oven material parameters and radiation model. The numerical calculations employ the SIMPLEC coupling algorithm^22,23^. The convective terms in the velocity equation and the k-equation are discretized using second-order upwind differencing in the three coordinate directions, while the diffusive terms are discretized using second-order central differencing.

Table 1 shows the setting of oven boundary conditions. Due to the convection heat transfer caused by the contact between the oven door glass and air and the radiation generated by the internal heater of the oven, Mixed mode was selected for the door wall. The bottom of the oven is in contact with the ground, and Radiation heat transfer is the main heat transfer mode. The enamelled domains (The oven cavity, the fan cover and the baking tray) are considered as the composite materials of steel and enamel.

### Mesh analysis

**Figure 4:**
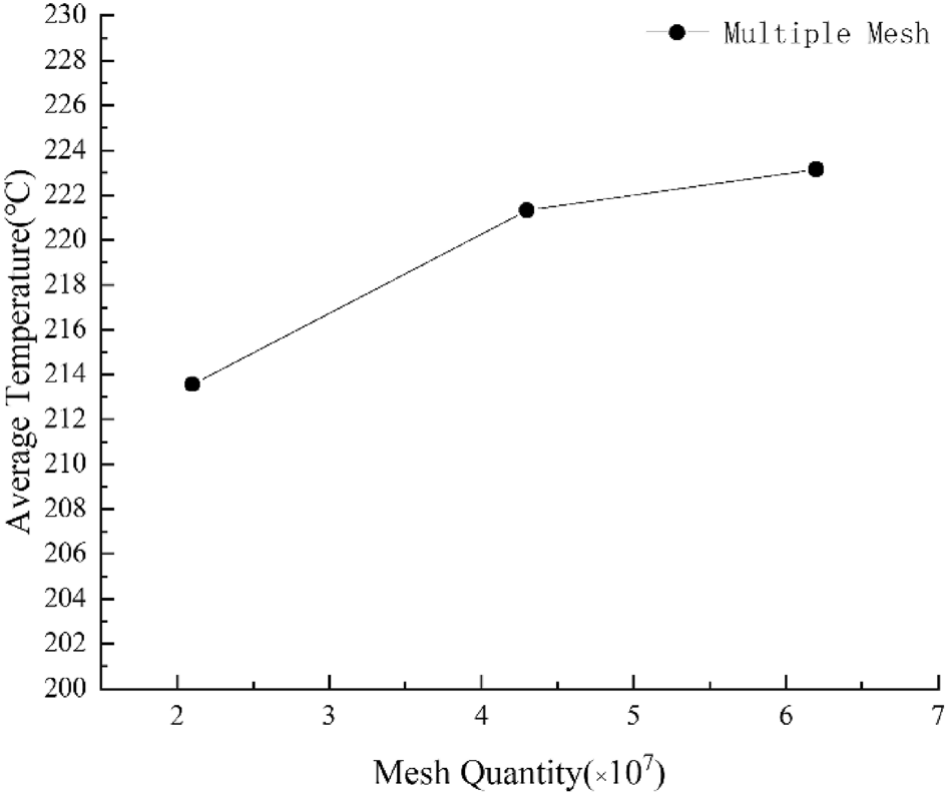
Grid independence verification.

Tetrahedral mesh element is used to divide the computing domain. The heating tube, fan cover, glass door and the volume grid at the fan are encrypted. By comparing the simulation results of 2.1 × 10^7^, 4.3 × 10^7^, 6.2 × 10^7^, the influence of grid on computer simulation precision was studied. A steady-state simulation was carried out for a no-load oven, and 13 monitoring points were placed in the center plane. After the simulation convergence, the data of 13 monitoring points were taken and averaged. By comparing the temperature data of three examples with different number of grids, it is found that 2.1 × 10^7^ is far from the other two simulation results. Since the simulation results of 4.3 × 10^7^ mesh and 6.2 × 10^7^ mesh was not different, we chose 4.3 × 10^7^ mesh density for further simulation. Independence verification is shown in Fig. 4.

## Equations and mathematical expressions

### Numerical simulation accuracy verification

For the initial oven structure, the internal cavity temperature was measured and compared with the numerical simulation results. As shown in Table 2, the numerical simulation results are in good agreement with the observed temperature distribution trend.

**Table 2:** Comparison of temperature data between simulation and experiment.

Simulation temperature			Experimental temperature		
P1:198.4	P2:199.2	P3:194.5	P1:196.7	P2:191.1	P3:197.2
P4:199.4	P5:202.0	P6:194.6	P4:200.9	P5:201.2	P6:198.8
P7:195.3	P8:201.5	P9:191.7	P7:200.4	P8:198.4	P9:196.7
P10:197.6	P11:196.3	P12:198.4	P10:199.0	P11:198.2	P12:200.3
P13:199.1			P13:199.5		

The formula used to evaluate the consistency of simulation data is:

$$e_{abs} = {{100} \mathord{\left/ {\vphantom {{100} n}} \right. \kern-0pt} n}\sum\limits_{i = 1}^{n} {\left( {{{\left( {T_{e} - T_{p} } \right)} \mathord{\left/ {\vphantom {{\left( {T_{e} - T_{p} } \right)} {T_{e} }}} \right. \kern-0pt} {T_{e} }}} \right)} i$$

where, $e_{abs}$ is the mean absolute error, %; *n* is the number of temperature measuring points in the measuring range; *T*_*e*_ is the experimental test temperature value, °C; *T*_*p*_ is the temperature value obtained by numerical simulation, °C.

**Figure 5:**
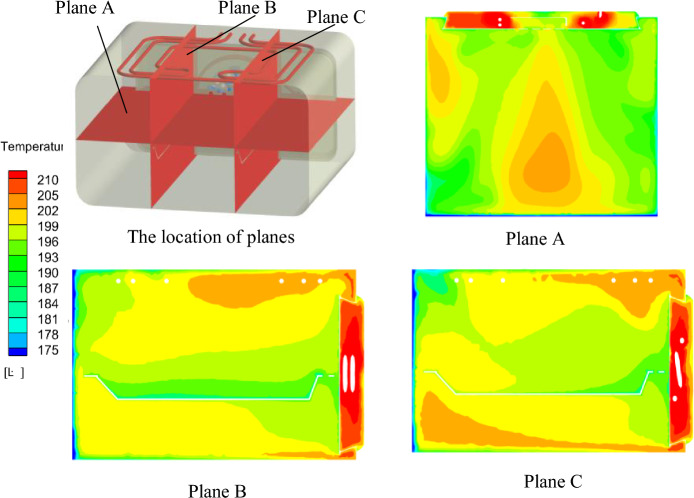
The contour of temperature in YZ surface.

**Figure 6:**
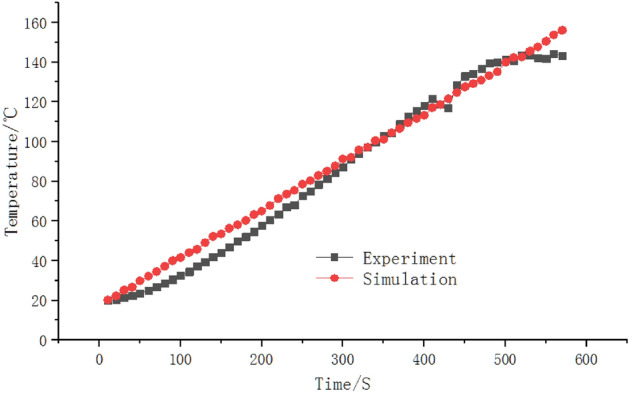
Comparison of temperature between experimental values and simulation results.

The average simulated temperature in the oven cavity is 195.4 °C, and the test temperature is 197.7 °C. The simulated value is lower than the measured value at 2.3 °C. As can be seen from Fig. 5, the temperature of the inner cavity of the oven near the wall is higher, while the temperature near the oven door body is lower. The temperature of the inner cavity from the back wall of the oven to the oven door body is decreasing gradually. Both the simulated and measured results show that the temperature field inside the oven is significantly uneven, and the standard deviation of temperature is 4.63. Figure 6 shows a comparison of simulation and actual measurement.

### Velocity distribution characteristics and optimization scheme

#### Velocity field distribution

In the air baking mode, the rear heating tube is activated with a total power of 1300 W, while the rear centrifugal fan is turned on, providing an additional power of 20 W. The fan operates at a speed of 1300 r·min^−1^ and has fan blades with a radius of 63 mm. Its primary purpose is to enhance convection effects within the oven. This mode is specifically designed to meet the high-temperature requirements of meat-based foods and employs a dedicated rotisserie grill. It effectively ensures the food's coloration is uniform and minimizes the risk of uneven cooking.

**Figure 7:**
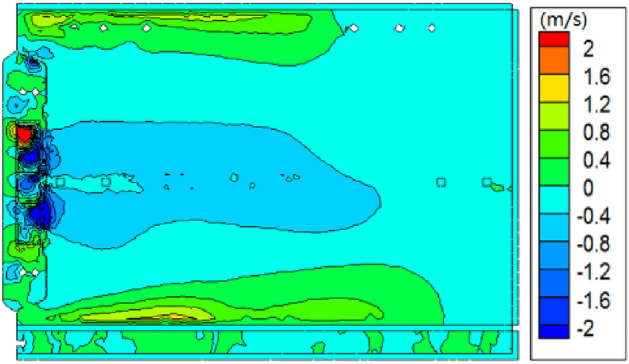
The contour of velocity in cavity.

**Figure 8:**
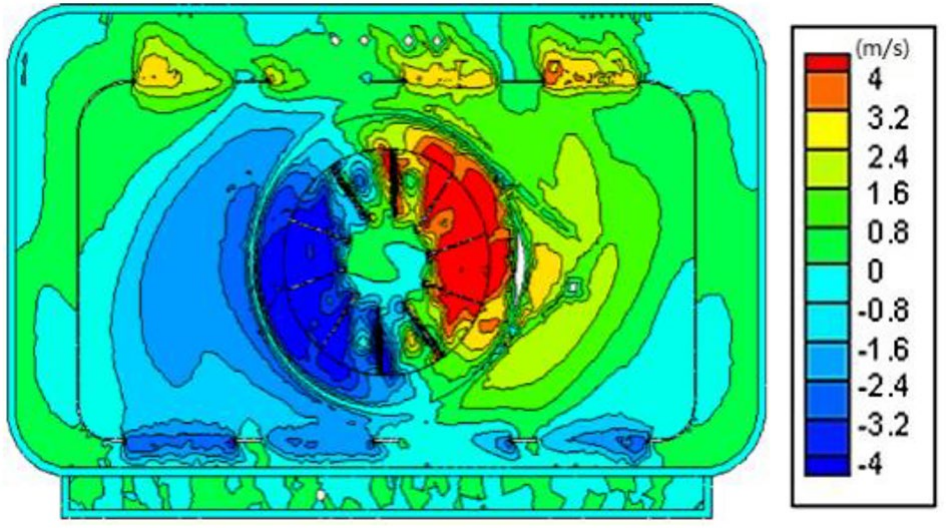
The contour of velocity in fan cover.

The numerical simulation results show that there are large-scale eddies inside the oven, as shown in Figs. 7 and 8. Because the center of the vortex is in the dead zone of flow, the convection heat transfer effect is poor in the actual heating process, so the temperature in this area is significantly lower than that in the surrounding area. The central vortex of the oven is caused by the large circumferential velocity component at the fan outlet, so eliminating the vortex requires reducing the circumferential velocity component at the fan outlet. The loss of momentum at the hot air outlet of the original oven is relatively serious, which causes the wind speed to decrease when the hot air enters the main flow field, thus reducing the heating effect of the hot fan. The Fig. 8 shows that the main reason is that the original cover around the outlet side close to the wall, and the small opening, the upper and lower side wind far away from the wall, and the opening is bigger, lead to hot air directly after a fan speed collision enclosure wall, the larger hot blast momentum loss, so the outlet by improving the baffle structure can improve local irregularity inside the oven.

#### Optimization scheme

The author sorts out a set of calculation formulas. The comparison between the simulation data and the calculated data shows that the results are consistent, as shown in Fig. 6.

**Figure 9:**
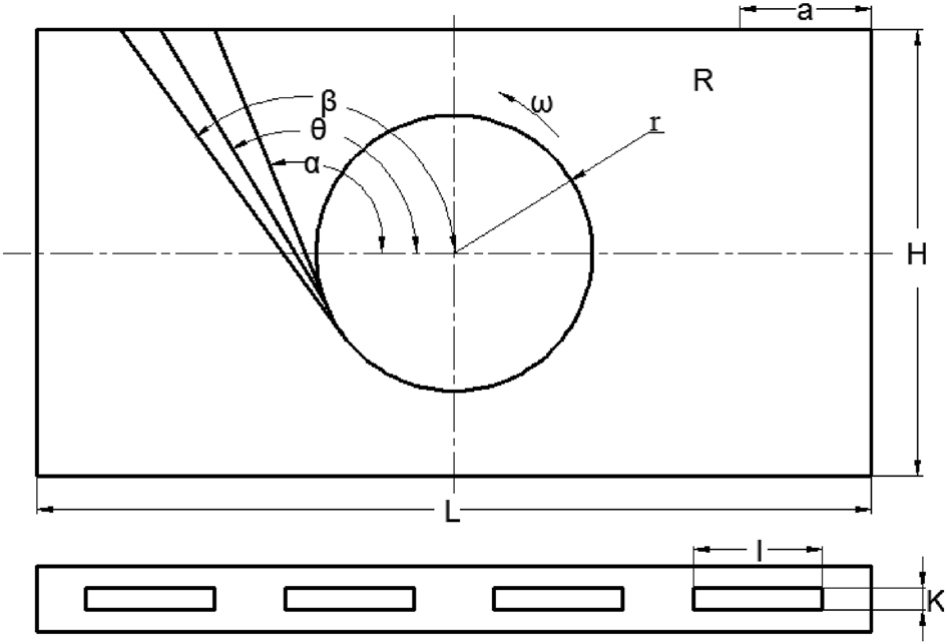
Schematic diagram of air volume distribution formula.

The Schematic diagram of air volume distribution formula is shown in Fig. 9. The air volume distribution formula is arranged as follows:

$$Q = klv\left( {\cos \alpha - \cos \beta } \right)$$

$$\alpha = \arctan \frac{H}{L} + \arcsin \frac{r}{{\sqrt {H^{2} + L^{2} } }}$$

where, H = 102 mm, indicating half the height of the baffle; T = 190.5 mm, indicating half the length of the baffle; K = 10 mm represents the thickness of the baffle opening; *V* = 8.57 m/s, indicating the edge velocity of the fan impeller; *α* means the starting Angle, *β* means the ending Angle; L1 represents the starting position of the opening; L2 represents the end position of the opening; L = L2-L1, length of air outlet; The fan radius is r.

**Figure 10:**
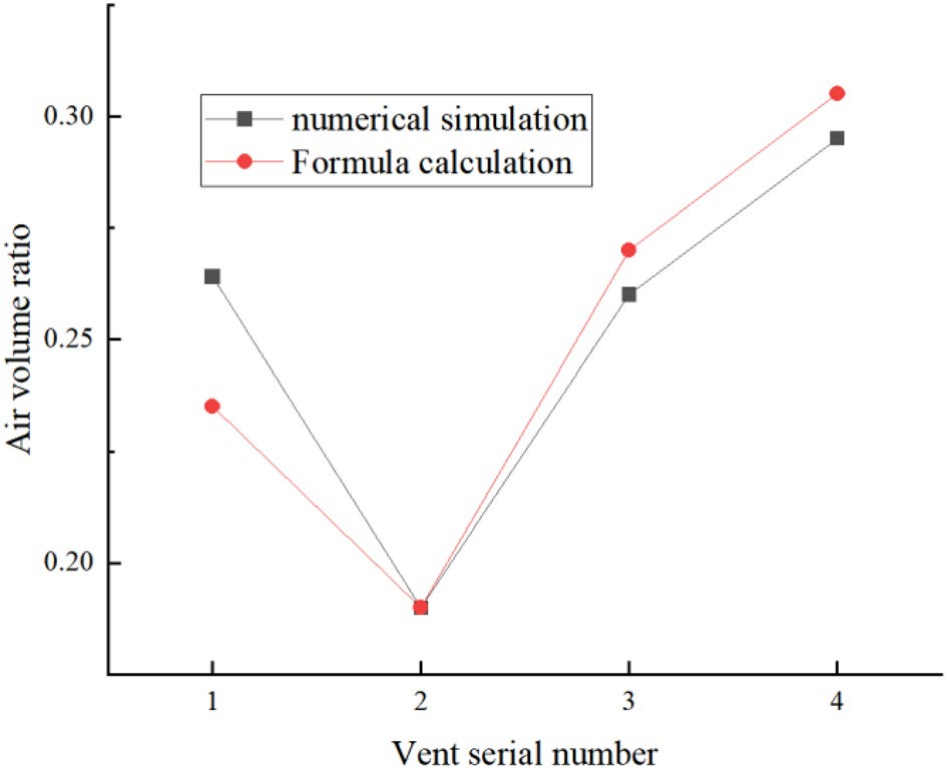
Comparison between numerical simulation and formula calculation data.

Figure 10 depicts a comparative analysis of data between numerical simulation and formula calculation for a fan speed of 600 r/min and an equivalent diameter of 16.67 mm. The graph clearly demonstrates a close resemblance between the results obtained from numerical simulation and those derived from the formula, validating the reliability of the formula. This discovery signifies that the formula holds significant guiding implications for design work in real-world engineering applications.

Based on the above analysis, it is evident that the airflow distribution at the outlet is uneven for the original baffle structure, even when the outlet size remains constant. This leads to a significant temperature disparity in the forced hot air, exacerbating the non-uniform temperature trend within the oven^24–26^. To address this issue, we made modifications by adjusting the length of the baffle outlet at different positions while keeping the width unchanged. The aim was to achieve a similar proportion of airflow from each outlet on the same side, relative to the total airflow.

**Figure 11:**
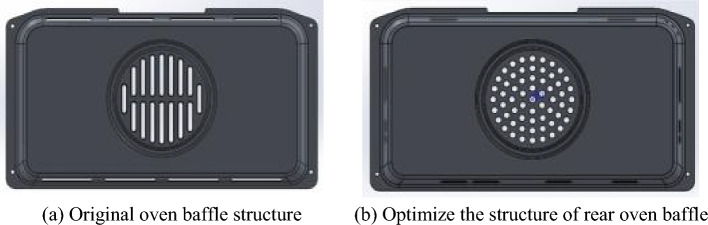
Structure comparison of oven baffle before and after optimization.

According to the flow field simulation results under the air baking mode of the oven, the air baffle was improved as follows. The upper section of the original baffle was removed, leaving only the intake holes on the left and right outer sides. Additionally, three small circular holes with a diameter of 6 mm were added on the right shoulder of the baffle to increase the outlet air pressure. This modification ensured that the hot air was directed towards the area closest to the glass door, thereby raising the temperature near the glass door. Furthermore, the right side of the baffle was equipped with a long hole and a pair of short holes to balance the airflow and achieve a more uniform temperature distribution inside the oven. The number of holes on the lower section of the baffle remained the same, but their sizes were reduced. A comparison of the baffle structures before and after the improvements is illustrated in Fig. 11.

In the air baking mode, the temperature measurement data before and after the improvement of the oven are shown in Table 3.

**Table 3:** Comparison of temperature measurement data of two baffles.

Fan cover plate structure	Working temperature (°C)	Maximum temperature difference (°C)	RMS (9 points)
Original structure	200	29	7.0728
Original structure	230	29	7.3182
Improved structure	150	8	2.9355
Improved structure	200	14	4.7402
Improved structure	250	18	5.7323

Based on the aforementioned data, it is evident that the improved baffle exhibits superior temperature uniformity compared to the original baffle. In the air baking mode, prior to program optimization, the maximum temperature difference of the original fan cover plate was 29 °C at 200 °C and 230 °C. Under the working temperatures of 150 °C, 200 °C, and 250 °C, the improved structure in the air baking mode displayed maximum temperature differences of 8 °C, 14 °C, and 18 °C, respectively. These three sets of data indicate an increasing trend in the maximum temperature difference with higher working temperatures. It can be concluded that the maximum temperature difference of the optimized original structure fan cover plate should be less than 29 °C when operating at 200 °C, underscoring the effectiveness of the program optimization in enhancing the temperature uniformity of the oven. Furthermore, the temperature field uniformity of the improved baffle, as determined by the root mean square (RMS) values, is notably superior to that of the original baffle within the oven.

## Conclusions

Based on the application of numerical simulation analysis methods, this study aims to investigate the internal airflow and associated heat transfer characteristics in a forced convection oven system. Building upon this foundation, it conducts a comprehensive analysis of the temperature distribution uniformity features, identifies the primary factors contributing to uneven temperature field distribution, and proposes effective solutions tailored to practical requirements.

The post-heating system is a crucial component of the oven. Through in-depth analysis of the oven, we have derived relevant formulas for the distribution of air volume in the hole layout of the rear baffle. Based on the principles of airflow distribution, we have designed and tested an improved baffle scheme. The results indicate that the improved baffle can significantly improve temperature uniformity compared to the original baffle. The derived theoretical formula for airflow distribution can be applied in practical engineering and provides valuable guidance for optimizing testing and development of ovens.

## Data Availability

The datasets used or analyses during the current study are available from the corresponding author on reasonable request.
